# NRF2‐CDH6‐CEBPD axis suppresses ferroptosis and drives cisplatin resistance in non‐small cell lung cancer

**DOI:** 10.1002/ccs3.70115

**Published:** 2026-09-23

**Authors:** Guang‐ze Zhou, Zhuo‐hang Zhu, Hong‐li Liao, Rui‐heng Chen, Dan‐ni Wu, Ri‐sheng Huang

**Affiliations:** ^1^ Department of Thoracic Surgery Wenzhou Central Hospital Affiliated to Wenzhou Medical University Wenzhou China; ^2^ Department of Pathology Wenzhou Central Hospital Affiliated to Wenzhou Medical University Wenzhou China

**Keywords:** CDH6, chemotherapy, drug resistance, ferroptosis, lung cancer

## Abstract

Ferroptosis is a non‐apoptotic form of programmed cell death driven by iron‐dependent lipid peroxidation. Induction of ferroptosis has been shown to overcome chemoresistance in cancer cells. Given the importance of the nuclear factor erythroid 2‐related factor 2 (NRF2) pathway in the regulation of cellular oxidative stress, this study was designed to search for NRF2‐regulated genes involved in ferroptosis and chemoresistance in non‐small cell lung cancer (NSCLC). Bioinformatics analysis and experimental validation indicated cadherin‐6 (CDH6) as a candidate gene. The expression and role of CDH6 in NSCLC were explored. CDH6 protein partners were identified and investigated after CDH6 co‐immunoprecipitation assays and mass spectrometry. Our data demonstrated that NRF2 binding to the promoter of the CDH6 gene inhibited RUNX2‐mediated transcription of CDH6 in NSCLC. Clinically, CDH6 was downregulated in NSCLC tissues and correlated with more advanced tumor‐node‐metastasis (TNM) stage and shorter overall survival. Knockdown of CDH6 enhanced the proliferation, colony formation, tumorigenesis, and cisplatin (CDDP) resistance in NSCLC cells. Moreover, overexpression of CDH6 induced ferroptosis and increased CDDP sensitivity in CDDP‐resistant NSCLC cells. Mechanistically, CDH6 associated with and sequestered CCAAT enhancer binding protein delta (CEBPD) protein in the cytoplasm and impaired the CEBPD‐dependent transcription of target genes. Depletion of *CEBPD* increased CDDP sensitivity of CDH6‐depleted NSCLC cells, whereas overexpression of CEBPD restored CDDP resistance in CDH6‐overexpressing NSCLC cells. Additionally, CEBPD upregulation was associated with poor prognosis in NSCLC patients. In conclusion, our data underscore the critical role of the NRF2‐CDH6‐CEBPD axis in modulating CDDP sensitivity in NSCLC and offer a potential therapeutic target for improving chemotherapy.

## Graphical abstract

Nuclear factor erythroid 2‐related factor 2‐mediated repression of cadherin‐6 (CDH6) expression is associated with poor prognosis in non‐small cell lung cancer (NSCLC). CDH6 induces ferroptosis and overcomes CDDP resistance in NSCLC cells by sequestering CEBPD protein in the cytoplasm.

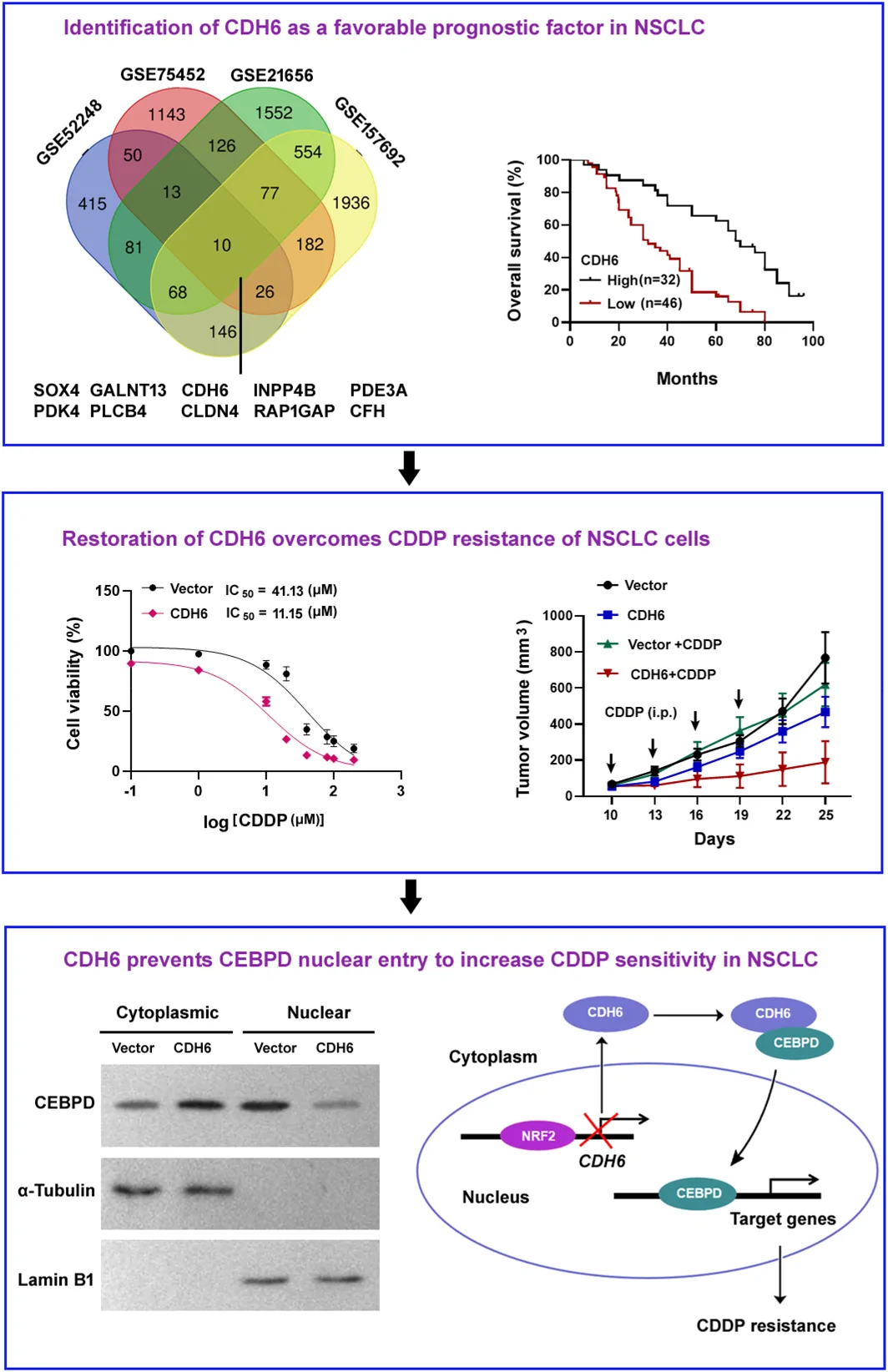

## 1 INTRODUCTION

Lung cancer is one of the most prevalent malignancies worldwide.^1^ Non‐small cell lung cancer (NSCLC) constitutes the major type (over 80%) of lung cancer and attracts much research attention.^2^ Platinum‐based chemotherapy is employed as a neoadjuvant treatment for NSCLC patients, leading to improved surgical outcomes.^3^ However, drug resistance eventually causes treatment failure and tumor relapse in NSCLC patients. Therefore, there is an urgent need to develop an effective strategy to prevent or reverse chemoresistance.

Ferroptosis is a non‐apoptotic form of programmed cell death induced by oxidative stress.^4^ Iron‐dependent lipid peroxidation is a hallmark of ferroptosis.^5^ Exposure to pro‐ferroptotic stimuli triggers the generation of excessive reactive oxygen species (ROS), which induces the peroxidation of polyunsaturated fatty acid‐containing phospholipids and causes lethal permeabilization of the plasma membrane. To counteract ferroptosis, several antioxidant defense systems, such as the nuclear factor erythroid 2‐related factor 2 (NRF2) pathway, are activated.^6^ The transcription factor NRF2 serves as a master regulator of the antioxidant response and can regulate many downstream target genes involved in redox homeostasis.^7^ Ferroptosis is implicated in various physiological and pathological processes, including cancer.^8^ Most intriguingly, chemotherapy‐resistant cancer cells are vulnerable to ferroptosis.^9^ Pharmacological induction of ferroptosis has been reported to overcome chemoresistance in multiple cancers.^10^, ^11^ For instance, quercetin, a natural flavonoid, impedes gastric cancer progression by inducing ferroptosis through the NRF2/GPX4 axis.^12^ Hence, identification of key ferroptosis regulators is important to develop novel strategies to improve chemotherapy against cancers.

Cadherin‐6 (CDH6) is a member of the cadherin (CDH) family that mediates calcium‐dependent cell adhesion.^13^ Several lines of evidence have revealed the involvement of CDH6 in tumor progression. CDH6 dysregulation is associated with prognosis in gastric cancer and glioma.^14^, ^15^ CDH6 exhibits the ability to regulate epithelial‐mesenchymal transition, invasion, and metastasis.^16^, ^17^ Despite these findings, the expression and function of CDH6 in NSCLC remains unclear.

In this study, we found that CDH6 was downregulated and predicted poor prognosis in NSCLC. The role of CDH6 in NSCLC cell growth and cisplatin (CDDP) resistance was uncovered. The mechanism by which CDH6 modulated the CDDP sensitivity of NSCLC was further investigated.

## 2 MATERIALS AND METHODS

### 2.1 Cell lines

Human NSCLC cell lines A549, H1299, and H460 were obtained from the American Type Culture Collection (ATCC). They were validated to be free of mycoplasma contamination. The authenticity of the cell lines was verified by short tandem repeat analysis. The cell lines have not been previously reported as misidentified or contaminated.

The cells were cultured in Dulbecco's Modified Eagle Medium supplemented with 10% fetal bovine serum (FBS; Sigma‐Aldrich). CDDP‐resistant cell lines (A549/CDDP and H1299/CDDP) were generated after exposure to increasing concentrations of CDDP, as described previously.^18^ The CDDP‐resistant cell lines were maintained in the presence of 5 μM CDDP.

### 2.2 Cell treatment and viability assessment

For activation of NRF2 signaling, NSCLC cells were treated with sulforaphane (5 μM; Selleck Chemicals) for 24 h.^19^ For assessment of CDDP sensitivity, NSCLC cells were treated with different concentrations of CDDP (Sigma‐Aldrich) for 72 h before viability analysis.^18^ For modulation of ferroptosis, NSCLC cells were treated with liproxstatin‐1 (Lip‐1; 1 μM), ferrostatin‐1 (Fer‐1; 2 μM), or RSL3 (1 μM) for 24 h.^20^ Lip‐1, Fer‐1, and RSL3 were obtained from Selleck Chemicals. Cell viability was measured using the 3‐(4,5‐dimethylthiazol‐2‐yl)‐2,5‐diphenyltetrazolium bromide (MTT) assay. MTT (Sigma‐Aldrich) solution was added to the cell culture and incubated in the dark for 4 h. Dimethyl sulfoxide was then added to each well. The absorbance of each well was measured at a wavelength of 570 nm. The half‐maximal inhibitory concentration (IC_50_) for CDDP was calculated.

### 2.3 Analysis of lipid peroxidation

Lipid peroxidation was evaluated by quantifying the lipid peroxidation product 4‐hydroxynonenal (4‐HNE).^21^ In this study, we used the Lipid Peroxidation (4‐HNE) Assay kit (Abcam) to measure the 4‐HNE levels. In brief, NSCLC cells were suspended in lysis buffer and added to a microplate precoated with 4‐HNE. The microplate was then incubated with anti‐4‐HNE antibody and horseradish peroxidase (HRP)‐conjugated secondary antibody. 3,3′,5,5′‐Tetramethylbenzidine was added as a substrate. The absorbance value was measured at 450 nm.

### 2.4 Lipid ROS production assessment

For analysis of lipid ROS production, C11‐BODIPY (581/591) (#D3861, Invitrogen) was used. Briefly, 2 μM C11‐BODIPY was added to the cell culture and incubated for 30 min at 37°C. Then, the cells were analyzed using a flow cytometer.

### 2.5 Bioinformatics analysis

The gene expression profile datasets GSE52248, GSE75452, GSE157692, and GSE21656 were retrieved from the Gene Expression Omnibus (GEO) database. We re‐analyzed the RNA sequencing data of GSE52248 (6 normal lung tissues and 6 invasive lung cancer tissues), GSE75452 (NRF2 knockdown vs. control A549 cells) and GSE157692 (CDDP‐resistant vs. parental A549 cells) and the microarray data of GSE21656 (CDDP‐resistant vs. parental H460 cells). Differentially expressed genes (DEGs) were separately identified from the 4 GEO datasets using the GEO2R web tool (https://www.ncbi.nlm.nih.gov/geo/geo2r/). The criteria for identifying DEGs were adjusted *p*‐value <0.05 and fold change >2. Common DEGs were visualized using a Venn diagram. Additionally, the TFBIND tool (https://tfbind.hgc.jp/) was used to predict the transcription factor binding sites in the promoter region of the *CDH6* gene.

### 2.6 Quantitative real‐time polymerase chain reaction analysis

Total RNA was extracted from cell lines using the RNeasy Mini kit (Qiagen) following the manufacturer's instructions. cDNA synthesis was conducted using the iScript cDNA Synthesis kit (Bio‐Rad). Quantitative real‐time polymerase chain reaction (qPCR) analyses were performed using the SYBR Premix Ex Taq II kit (TaKaRa). The primer sequences are shown in Supplementary Table S1. The mRNA expression of each gene was determined using the 2^−ΔΔCt^ method and normalized to the reference gene *GAPDH*.

### 2.7 Patient samples and immunohistochemistry

A total of 78 NSCLC and adjacent normal lung tissue samples were obtained from NSCLC patients who received primary tumor resection. No patients had a history of preoperative anticancer treatments. Clinical information was retrieved from medical records.

Tissue sections were incubated with anti‐CDH6 (1:100 dilution; Abcam), anti‐CEBPD (1:100 dilution; Abcam), or anti‐NRF2 (1:100 dilution; Abcam) antibody at 4°C overnight. Staining was detected using the EnVision Dako system (Dako). The sections were counterstained with hematoxylin. Quantitative analysis of immunohistochemical staining was performed based on staining intensity and percentages of stained cells. The results are expressed as *H*‐scores (ranging between 0 and 300), which were calculated as 1 × % weak staining (1+) + (2 × % moderate staining (2+)) + (3 × % strong staining (3+)). Based on the median CDH6 or CCAAT enhancer binding protein delta (CEBPD) *H*‐scores, our NSCLC patients were divided into high and low expression groups.

### 2.8 Transient gene overexpression and knockdown

For overexpression studies, cDNAs encoding *CDH6* (NM_004932), *CEBPD* (NM_005195), or Runt‐related transcription factor 2 (*RUNX2*) (NM_001024630) were cloned into the pcDNA3.1(+) vector. The plasmids were transfected into NSCLC cells using Lipofectamine 3000 reagent (Invitrogen) according to the manufacturer's instructions. For transient gene silencing, a pool of 3 small interfering RNAs (siRNAs) targeting *NRF2* or *CEBPD* was transfected into NSCLC cells using Lipofectamine RNAiMAX reagent (Invitrogen) at a concentration of 40 nM. The transfected cells were incubated for 48 h before further analysis. The siRNA sequences are listed in Table S1.

### 2.9 Stable cell line generation

To produce stable knockdown cell lines, 2 different short hairpin RNAs (shRNAs) targeting *CDH6* mRNA were synthesized and cloned into the pLKO.1 puro vector. The *CDH6* targeting sequences were 5′‐CATACGACTCCTTGGCCACTT‐3′ (shCDH6#1) and 5′‐GCAACCACAAACCTAGTACGA‐3′ (shCDH6#2). The specific shRNA expression vector was transfected into NSCLC cells using Lipofectamine 3000 reagent, followed by selection of stable cell clones in the presence of 1 μg/mL puromycin (Sigma‐Aldrich).

### 2.10 Chromatin immunoprecipitation

Cells were cross‐linked with 1% formaldehyde for 15 min and lysed in chromatin immunoprecipitation (ChIP) Lysis Buffer (Abcam). Chromatin was sonicated to generate fragments of approximately 400 bp. After preclearing with control IgG, ChIP was performed using anti‐NRF2 antibody (Abcam) or control IgG. Protein G agarose beads were added and incubated for 2 h. Precipitated DNA was extracted and quantified using qPCR analysis. ChIP primers were as follows: forward, 5′‐GAGTTTAATTTGAAGGTTATC‐3′ and reverse, 5′‐TCCTGAGATGACAATACCGAT‐3′.

### 2.11 Luciferase reporter assay

The human *CDH6* promoter was cloned into the pGL3‐Basic Vector (Promega). NSCLC cells were plated in 24‐well plates (5 × 10^4^ cells per well) and transfected with the reporter plasmids along with the constructs expressing RUNX2. Sulforaphane (5 μM) was added to promote NRF2 activation. A plasmid pRL‐TK expressing *Renilla* luciferase was cotransfected to control for transfection efficiency. After 24 h, cells were tested for luciferase activity using the Dual‐Luciferase Reporter Assay System (Promega).

### 2.12 Western blot analysis

For preparation of whole‐cell lysates, NSCLC cells were lysed in radioimmunoprecipitation assay (RIPA) buffer supplemented with a protease inhibitor cocktail (Sigma‐Aldrich). For preparation of nuclear and cytoplasmic fractionation, the nuclear and cytoplasmic extraction kit (Thermo Fisher Scientific) was used. The protein samples (approximately 40 μg protein per lane) were separated in sodium dodecyl sulfate‐polyacrylamide gels and transferred onto polyvinylidene fluoride membranes. After blocking in 5% fat‐free milk, the membranes were incubated with anti‐CDH6 (1:1000 dilution; Abcam), anti‐RUNX2 (1:1000 dilution; Abcam), anti‐CEBPD (1:1000 dilution; Abcam), anti‐α‐tubulin (1:2000 dilution; Abcam), anti‐lamin B1 (1:2000 dilution; Abcam), anti‐GPX4 (1:1000 dilution; Abcam), anti‐ACSL4 (1:1000 dilution; Abcam), and anti‐GAPDH (1:2000 dilution; Abcam) antibodies overnight at 4°C. Then, the membranes were incubated with HRP‐labeled secondary antibodies for 1 h. The membranes were visualized using an enhanced chemiluminescence system (Thermo Fisher Scientific).

### 2.13 Cell proliferation assay

NSCLC cells transfected with the indicated constructs were seeded into 96‐well plates and cultured for 1–4 days. Cell viability was determined at indicated time points by the MTT method, and the proliferation curves were plotted.

### 2.14 Colony formation assay

A total of 500 cells were plated in each well of 6‐well plates. The cells were incubated for 14 days and stained with crystal violet. The colony number of each well was recorded.

### 2.15 Tumor formation in nude mice

Four‐week‐old male BALB/c nude mice were maintained under specific pathogen‐free conditions for 1 week before the experiment. For the establishment of a subcutaneous tumor model, CDH6‐depleted or control A549 cells were injected into the BALB/c nude mice (2 × 10^6^ cells per mouse). The volume of subcutaneous tumors was measured at 5, 10, 15, 20, 25, and 30 days after injection, and the tumor growth curve was then plotted. To assess the effect of CDH6 overexpression on CDDP sensitivity in vivo, CDH6‐overexpressing or control A549 cells were subcutaneously injected into nude mice (2 × 10^6^ cells per mouse). When tumors reached approximately 80 mm^3^ (10 days after cell injection), mice were randomly divided into 4 groups (*n* = 5): vector, CDH6, vector + CDDP, and CDH6 + CDDP. CDDP (2 mg/kg) was delivered via intraperitoneal injection (i.p.) once every 3 days for 4 times.^22^ Tumor volume was measured at indicated time points. After the last measurement, the mice were euthanized. The xenograft tumors were harvested and photographed.

### 2.16 Co‐immunoprecipitation assay and mass spectrometry

Total protein was extracted from NSCLC cells lysed in RIPA buffer containing the protease inhibitor cocktail. The protein samples were incubated with anti‐CDH6 or anti‐CEBPD antibody (Abcam) overnight at 4°C. The next day, Protein A + G agarose beads (Thermo Fisher Scientific) were added and incubated at 4°C for 4 h. The supernatant was discarded after centrifugation. The bead‐bound proteins were subjected to Western blot analysis or mass spectrometry.

For preparation of mass spectrometry samples, bead‐bound proteins were reduced with 5 mM dithiothreitol (Sigma‐Aldrich), alkylated with 10 mM iodoacetamide (Sigma‐Aldrich), and digested with trypsin (Sigma‐Aldrich). After digestion for 16 h, the peptide mixture was analyzed by a liquid chromatography‐tandem mass spectrometry detection system. The original mass spectrometry data were processed using Proteome Discoverer 2.1 (Thermo Fisher Scientific). The UniProt database (*Homo sapiens*) was used as a reference database for protein identification at a 1% false discovery rate. The proteins for which at least 2 unique peptides were detected were selected as candidates and subjected to further analysis.

### 2.17 Immunofluorescence staining

NSCLC cells were seeded on coverslips and cultured for 24 h. The cells were fixed with 4% paraformaldehyde and permeabilized with 0.1% Triton X‐100. The primary antibody against CEBPD (Abcam) or CDH6 (Abcam) was used and incubated overnight at 4°C, which was followed by incubation with secondary antibodies conjugated with Alexa Fluor 488 or Alexa Fluor 594 (Abcam) for 1 h. Cell nuclei were stained with 4′,6‐diamidino‐2‐phenylindole solution (Abcam). The stained cells were observed under a fluorescence microscope.

### 2.18 Statistical analysis

Data are expressed as mean and standard deviation. Two‐tailed Student's *t*‐test was used to compare the differences between two groups. One‐way analysis of variance and post hoc Tukey tests were done to determine the differences among multiple groups. Correlation analysis was performed using the Pearson correlation test. For Kaplan–Meier survival curves, statistical differences were compared by the log‐rank test. Significant differences were considered when *p* < 0.05.

## 3 RESULTS

### 3.1 NRF2‐mediated suppression of CDH6 expression in NSCLC cells

**FIGURE 1:**
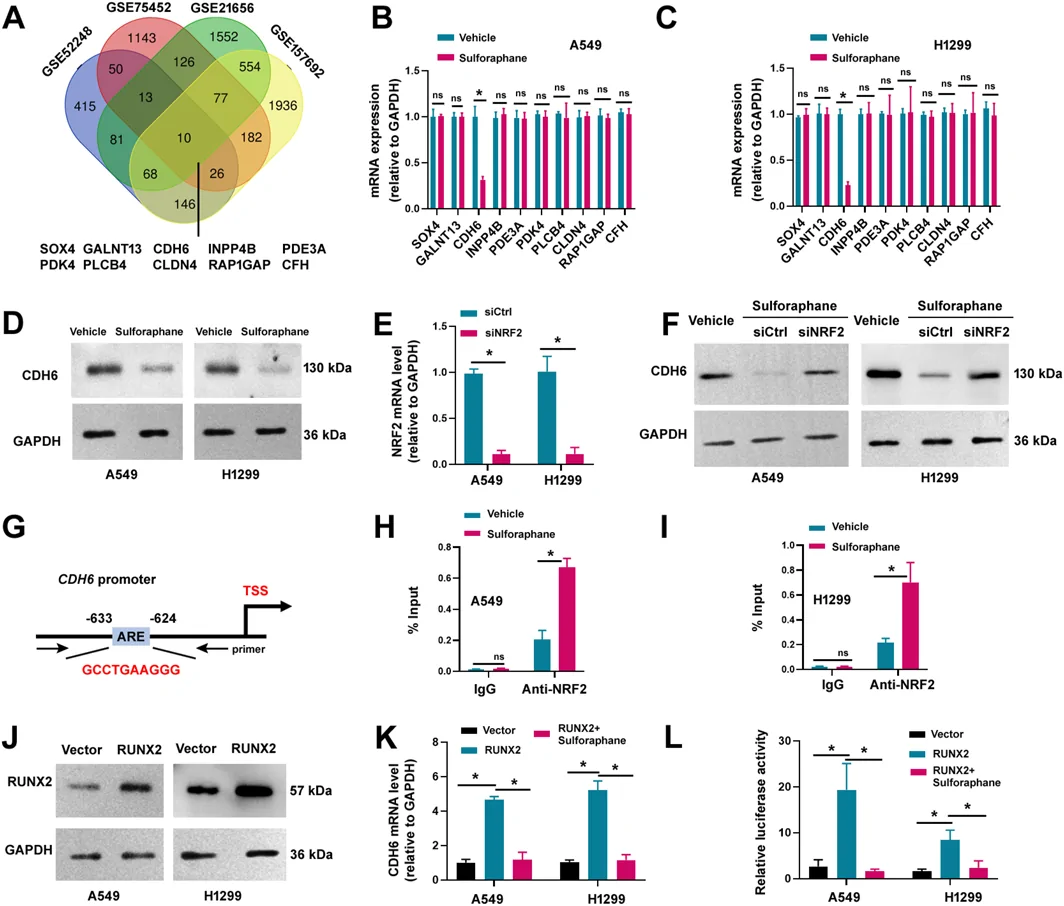
NRF2‐mediated suppression of CDH6 expression in NSCLC cells. (A) Venn diagram analysis showing 10 overlapping genes among the 4 Gene Expression Omnibus datasets: GSE52248 (6 normal lung tissues and 6 invasive lung cancer tissues), GSE75452 (*NRF2* knockdown vs. control A549 cells), GSE157692 (CDDP‐resistant vs. parental A549 cells) and microarray data from GSE21656 (CDDP‐resistant vs. parental H460 cells). (B, C) qPCR analysis was utilized to determine the mRNA levels of indicated genes after treatment with 5 μM sulforaphane. *p < 0.05 (*n* = 3). ns indicates no significance. (D) Western blot analysis was utilized for assessing CDH6 protein levels. (E) qPCR analysis was utilized to validate the NRF2 knockdown efficacy in A549 and H1299 cells transfected with control siRNA (siCtrl) or a pool of 3 NRF2‐targeting siRNAs (siNRF2). *p < 0.05 (*n* = 3). (F) Western blot analysis was utilized to determine the CDH6 protein levels. (G) Bioinformatic analysis predicts an antioxidant response element for NRF2 binding in the promoter region of the *CDH6* gene. TSS, transcriptional start site. (H, I) ChIP‐PCR assay was performed with anti‐NRF2 antibodies to evaluate NRF2 binding to the *CDH6* promoter. IgG was used as a negative control. *p < 0.05 (*n* = 3). ns indicates no significance. (J) RUNX2 overexpression was confirmed by Western blot analysis. (K) *CDH6* mRNA levels were measured by qPCR analysis. *p < 0.05 (*n* = 3). (L) NSCLC cells were transfected with luciferase reporter constructs alone or with the RUNX2‐expressing plasmid, and relative luciferase activity was determined 24 h later. *p < 0.05 (*n* = 3). CDDP, cisplatin; CDH6, cadherin‐6; NRF2, nuclear factor erythroid 2‐related factor 2; NSCLC, non‐small cell lung cancer; qPCR, quantitative real‐time polymerase chain reaction; RUNX2, Runt‐related transcription factor 2; siRNA, small interfering RNA.

The NRF2 pathway plays a central role in orchestrating oxidative stress response and survival in cancer cells.^23^ Thus, we attempted to search for key NRF2‐regulated genes involved in NSCLC chemoresistance based on publicly available transcriptomic data from the GEO database. Four GEO datasets (GSE52248, GSE75452, GSE157692, and GSE21656) were analyzed to identify DEGs. Specifically, the GSE52248 dataset showed the gene expression profiles between normal and lung cancer tissues, GSE75452 between control and NRF2‐silenced lung adenocarcinoma A549 cells, as well as GSE157692 and GSE21656 between parental and CDDP‐resistant NSCLC cells. A total of 10 common DEGs (*SOX4*, *GALNT13*, *CDH6*, *INPP4B*, *PDE3A*, *PDK4*, *PLCB4*, *CLDN4*, *RAP1GAP*, and *CFH*) were obtained for further analysis (Figure 1A). To validate the regulation of the candidate genes by NRF2, we treated 2 different NSCLC cells (A549 and H1299) with the NRF2 inducer, sulforaphane. Among the 10 genes analyzed, CDH6 mRNA expression was significantly inhibited by the sulforaphane treatment, but the other genes remained unchanged (Figure 1B,C). Western blot analysis confirmed that the protein level of CDH6 was markedly reduced in NSCLC cells after the sulforaphane treatment (Figure 1D). To exclude the off‐target effects of sulforaphane, we performed rescue experiments by silencing *NRF2* using specific siRNAs. The knockdown efficacy of *NRF2* in both A549 and H1299 cells was confirmed (Figure 1E). As expected, depletion of *NRF2* reversed sulforaphane‐mediated suppression of CDH6 expression (Figure 1F). Bioinformatic analysis suggested that the promoter region of the *CDH6* gene carried an antioxidant response element for NRF2 (Figure 1G). Notably, ChIP assays indicated that the sulforaphane treatment enhanced the enrichment of NRF2 at the promoter of *CDH6* (Figure 1H,I). A previous study has reported that the transcription factor RUNX2 is responsible for driving the transcription of *CDH6* in human thyroid tumors.^24^ Thus, we examined whether NRF2 occupancy interfered with RUNX2‐mediated transcription of *CDH6*. Of note, overexpression of RUNX2 increased the abundance of *CDH6* transcripts, which was blocked by the sulforaphane treatment (Figure 1J,K). Luciferase reporter assays further demonstrated that the luciferase activity under the control of the *CDH6* promoter was induced upon RUNX2 overexpression (Figure 1L). Such effect was compromised in the presence of sulforaphane. Taken together, these results suggest that NRF2 can repress the transcription of *CDH6* in NSCLC cells by binding to its promoter.

### 3.2 CDH6 downregulation predicts a poor prognosis of NSCLC patients

**FIGURE 2:**
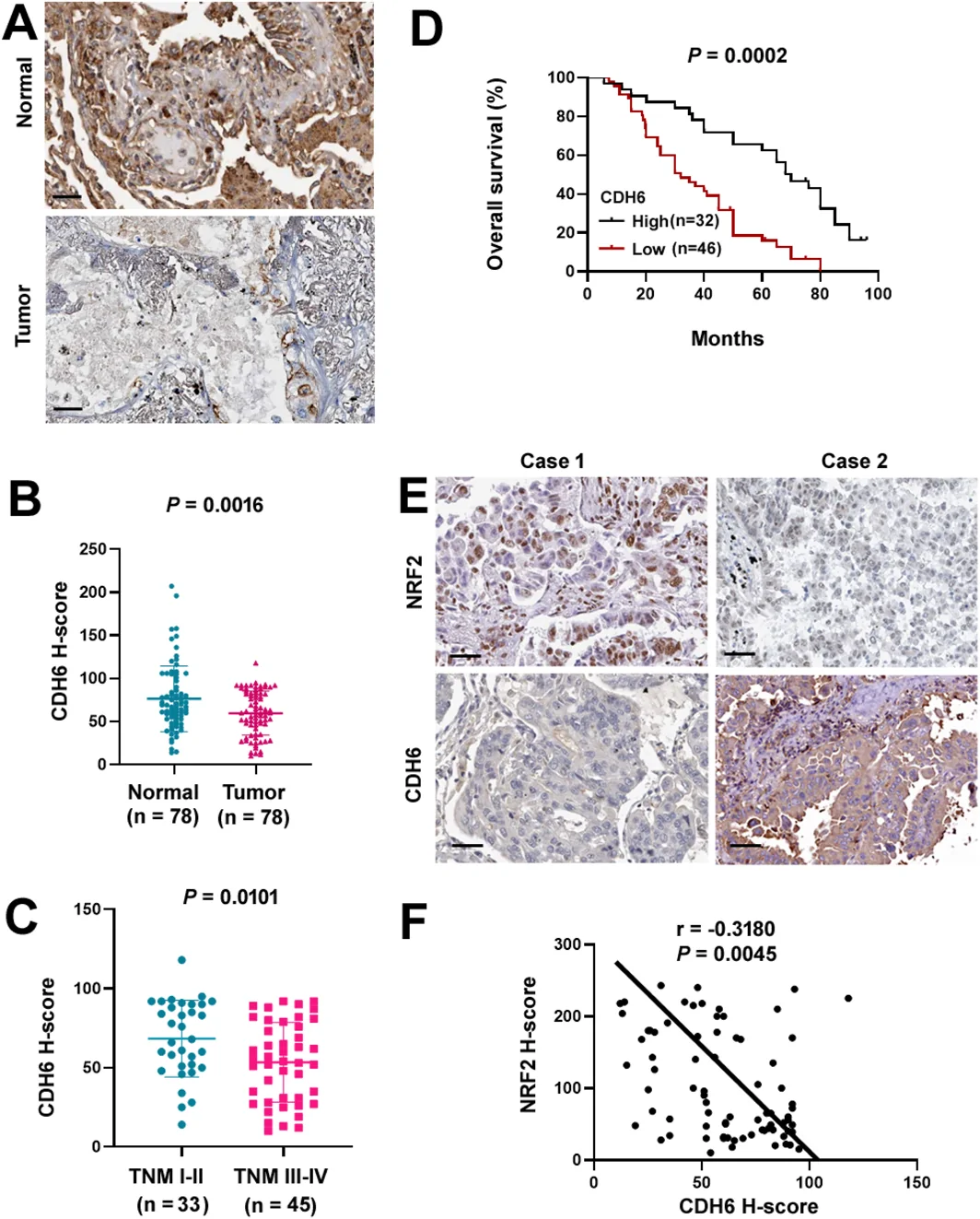
CDH6 downregulation predicts a poor prognosis in NSCLC patients. (A) Representative images of CDH6 immunohistochemical staining in NSCLC and adjacent normal lung tissues. Scale bar: 100 μm. (B) Comparison of *H*‐scores for CDH6 staining in NSCLC and adjacent normal lung tissues. (C) Comparison of *H*‐scores for CDH6 staining in NSCLC samples at different stages. (D) Kaplan–Meier curves of overall survival according to CDH6 expression levels (high vs. low). (E) Representative images of CDH6 and NRF2 immunohistochemical staining in two cases of NSCLC. Scale bar, 100 μm. (F) Correlation between CDH6 and NRF2 *H*‐scores in NSCLC samples. CDH6, cadherin‐6; NSCLC, non‐small cell lung cancer; NRF2, nuclear factor erythroid 2‐related factor 2.

To evaluate the clinical relevance of CDH6 in NSCLC, we performed immunohistochemical staining in a series of NSCLC and adjacent normal lung tissues (*n* = 78). Compared with adjacent lung tissues, NSCLC specimens exhibited reduced expression of CDH6 (*p* = 0.0016; Figure 2A,B). Low expression of CDH6 in lung tumors was significantly associated with more advanced TNM stage in NSCLC patients (*p* = 0.0101; Figure 2C). Moreover, the NSCLC patients with low tumoral expression of CDH6 had significantly shorter overall survival than those with high expression of CDH6 (*p* = 0.0002; Figure 2D). Additionally, we examined the association between CDH6 and NRF2 expression in NSCLC tissues. The immunohistochemical staining results indicated a negative correlation between CDH6 and NRF2 expression (*r* = −0.3180, *p* = 0.0045; Figure 2E,F). Our data suggest the involvement of CDH6 downregulation in NSCLC progression.

### 3.3 CDH6 deficiency promotes tumorigenesis and confers CDDP resistance in NSCLC cells

**FIGURE 3:**
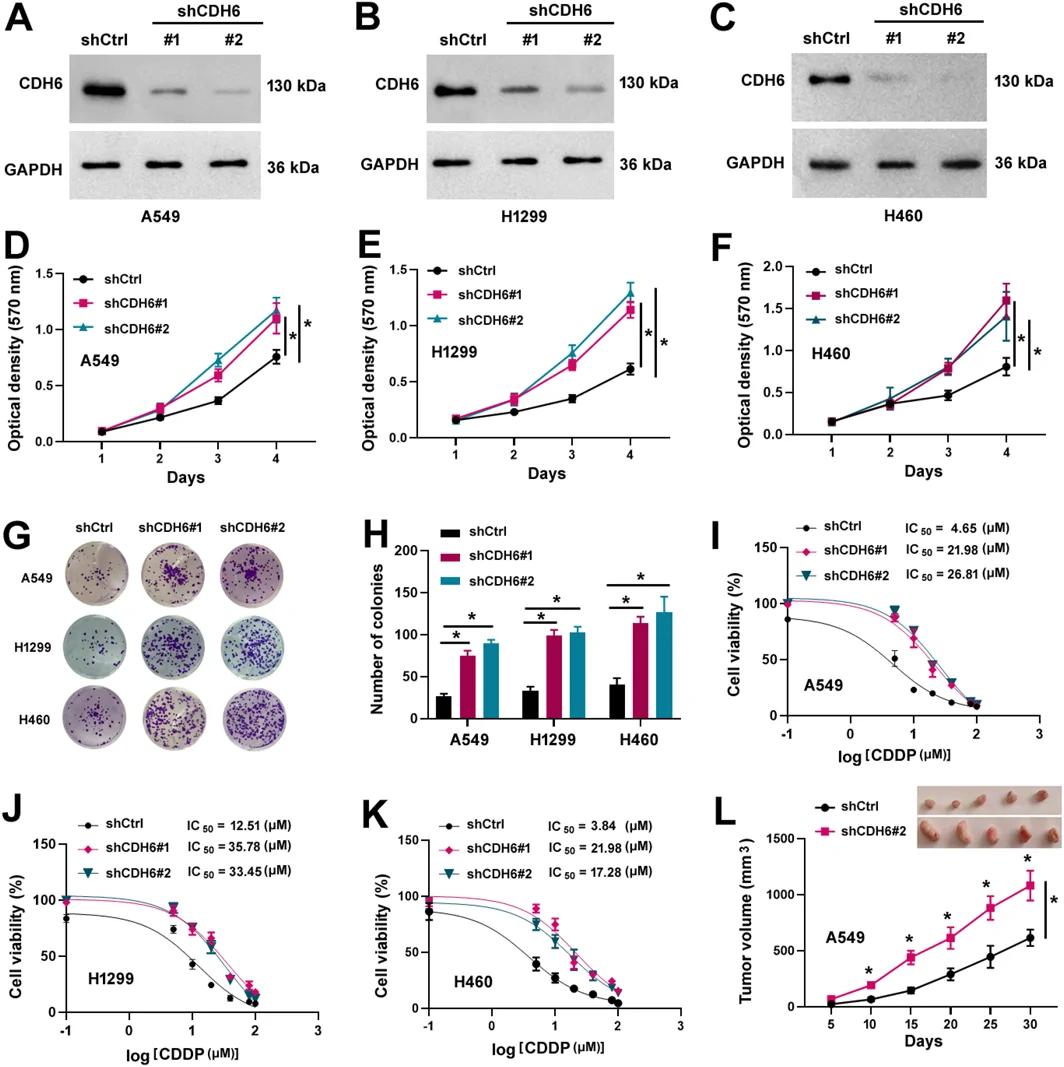
CDH6 deficiency promotes tumorigenesis and confers CDDP resistance in non‐small cell lung cancer cells. (A–C) Western blot analysis was utilized to determine the efficiency of CDH6 knockdown in A549, H1299, and H460 cells transfected with control shRNA (shCtrl) or CDH6‐targeting shRNA (shCDH6). (D–F) Cell proliferation was measured using the MTT assay. *p < 0.05 (*n* = 3). (G, H) Colony counting was conducted 14 days after seeding. *p < 0.05 (*n* = 3). (I–K) The IC_50_ of CDDP in A549 and H1299 cells was assessed by the MTT assay. (L) Tumor volume in nude mice was monitored every 5 days after cell injection (*n* = 5). *p < 0.05. Inserts: images of the xenograft tumors after dissection. CDDP, cisplatin; CDH6, cadherin‐6; MTT, 3‐(4,5‐dimethylthiazol‐2‐yl)‐2,5‐diphenyltetrazolium bromide; shRNA, short hairpin RNA.

Next, we explored the biological function of CDH6 in NSCLC cells. shRNA‐mediated knockdown of CDH6 was performed in A549, H1299, and H460 cells (Figure 3A–C). Compared to control cells, CDH6‐depleted NSCLC cells exhibited increased proliferation and colony formation capacities (Figure 3D–H). We also measured the survival of CDH6‐depleted NSCLC cells after exposure to different concentrations of CDDP. Intriguingly, CDH6 depletion reduced the cytotoxic effect of CDDP on NSCLC cells (Figure 3I–K). A xenograft tumor model was established in nude mice by subcutaneously injecting CDH6‐depleted A549 cells. Compared to control tumors, loss of CDH6 facilitated the growth of A549 xenograft tumors in nude mice (Figure 3L). These results indicate that CDH6 functions as a tumor suppressor in NSCLC.

### 3.4 Reconstitution of CDH6 promotes ferroptosis and restores CDDP sensitivity in CDDP‐resistant NSCLC cells

**FIGURE 4:**
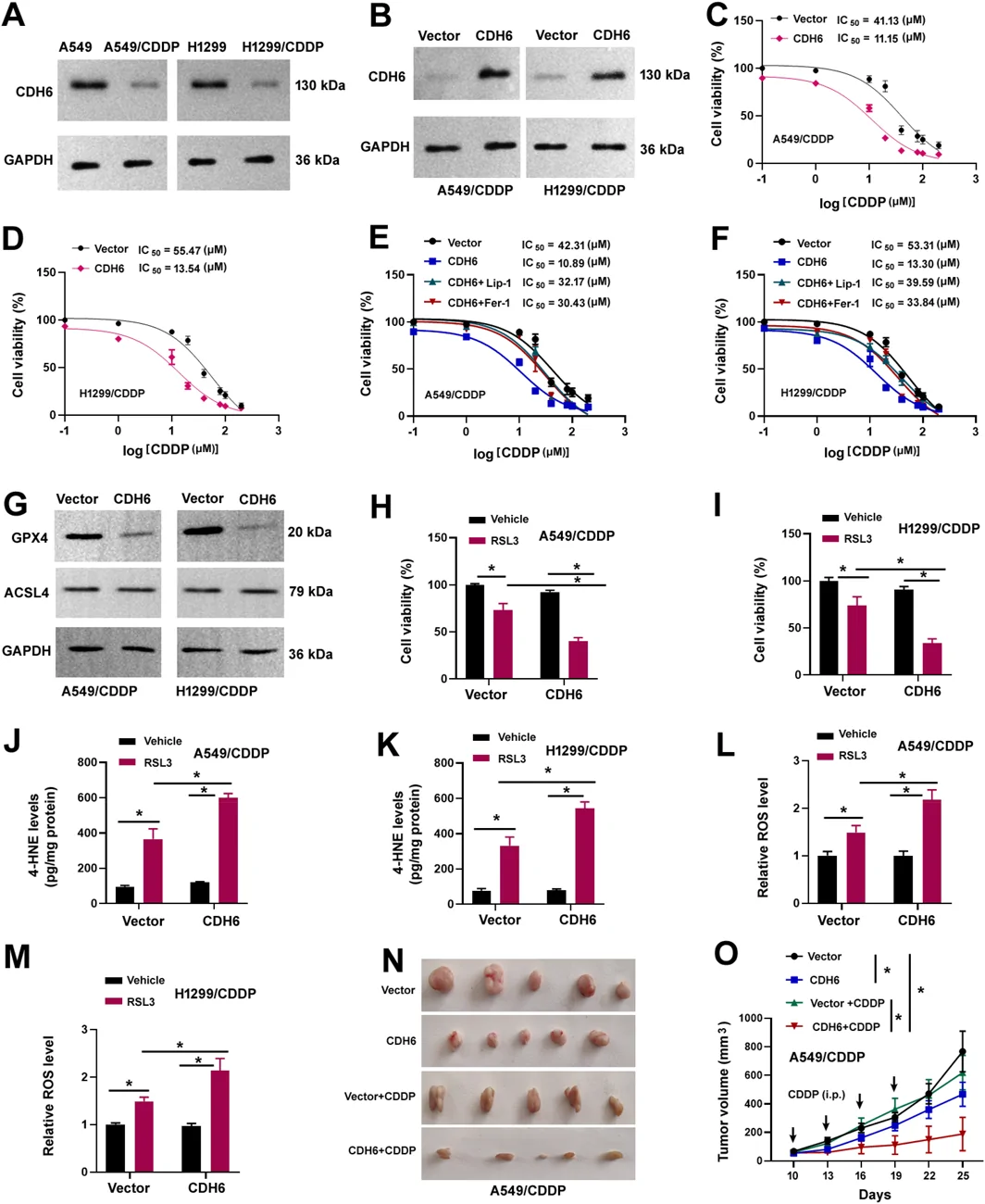
Reconstitution of CDH6 promotes ferroptosis and restores CDDP sensitivity in CDDP‐resistant NSCLC cells. (A) Western blot analysis of CDH6 in CDDP‐resistant (A549/CDDP and H1299/CDDP) and parental NSCLC cells. (B) Western blot analysis was utilized to validate the overexpression of CDH6 in CDDP‐resistant cells. (C, D) Measurement of the IC_50_ of CDDP in A549/CDDP and H1299/CDDP cells transfected with the indicated constructs. (E, F) Treatment with the ferroptosis inhibitors liproxstatin‐1 (Lip‐1) or ferrostatin‐1 (Fer‐1) affected the IC_50_ of CDDP in A549/CDDP and H1299/CDDP cells transfected with the indicated constructs. (G) Western blot analysis of ACSL4 and GPX4 protein levels. (H, I) Assessment of cell viability after treatment with the ferroptosis inducer RSL3. *p < 0.05 (*n* = 3). (J, K) Assessment of lipid peroxidation by quantifying the lipid peroxidation product 4‐hydroxynonenal levels. *p < 0.05 (*n* = 3). (L, M) Analysis of lipid reactive oxygen species production by flow cytometry using the C11‐BODIPY (581/591) probe. *p < 0.05 (*n* = 3). (N, O) Assessment of the role of CDH6 in modulating CDDP efficacy in vivo. CDDP (2 mg/kg) was delivered via intraperitoneal injection (i.p.) once every 3 days for 4 times. Representative images of the subcutaneous xenograft tumors after dissection are shown in panel (N). *p < 0.05 (*n* = 5). CDDP, cisplatin; CDH6, cadherin‐6; NSCLC, non‐small cell lung cancer.

To determine the role of CDH6 in CDDP resistance, we established CDDP‐resistant A549 and H1299 cells, which showed significantly higher IC_50_ values compared to their parental counterparts (Figure S1). Given that CDDP‐resistant NSCLC cells had lower levels of CDH6 than parental cells (Figure 4A), we checked whether reconstitution of CDH6 could overcome CDDP resistance in CDDP‐resistant NSCLC cells. As expected, overexpression of CDH6 significantly increased CDDP sensitivity in CDDP‐resistant A549 and H1299 cells (Figure 4B–D). Induction of ferroptosis is an important mechanism for improving chemosensitivity in cancer cells.^25^ Treatment with the ferroptosis inhibitors liproxstatin‐1 (Lip‐1) or ferrostatin‐1 (Fer‐1) dramatically rescued CDH6‐dependent sensitization of CDDP‐resistant NSCLC cells to CDDP (Figure 4E,F). Analysis of key ferroptosis regulators (ACSL4 and GPX4) revealed that CDH6 overexpression markedly reduced the expression of GPX4 rather than ACSL4 (Figure 4G). To substantiate the role of CDH6 in ferroptosis, we treated CDH6‐overexpressing cells with the ferroptosis inducer RSL3. Notably, enforced expression of CDH6 enhanced RSL3‐induced ferroptosis, lipid ROS production, and lipid peroxidation in CDDP‐resistant NSCLC cells (Figure 4H–M). We then assessed the role of CDH6 in modulating CDDP efficacy in vivo. We found that overexpression of CDH6 suppressed tumor growth and increased the sensitivity to CDDP (Figure 4N,O). Taken together, CDH6 overexpression attenuates CDDP resistance in NSCLC cells by potentiating ferroptosis.

### 3.5 CDH6 interacts with CEBPD and inhibits its nuclear localization

**FIGURE 5:**
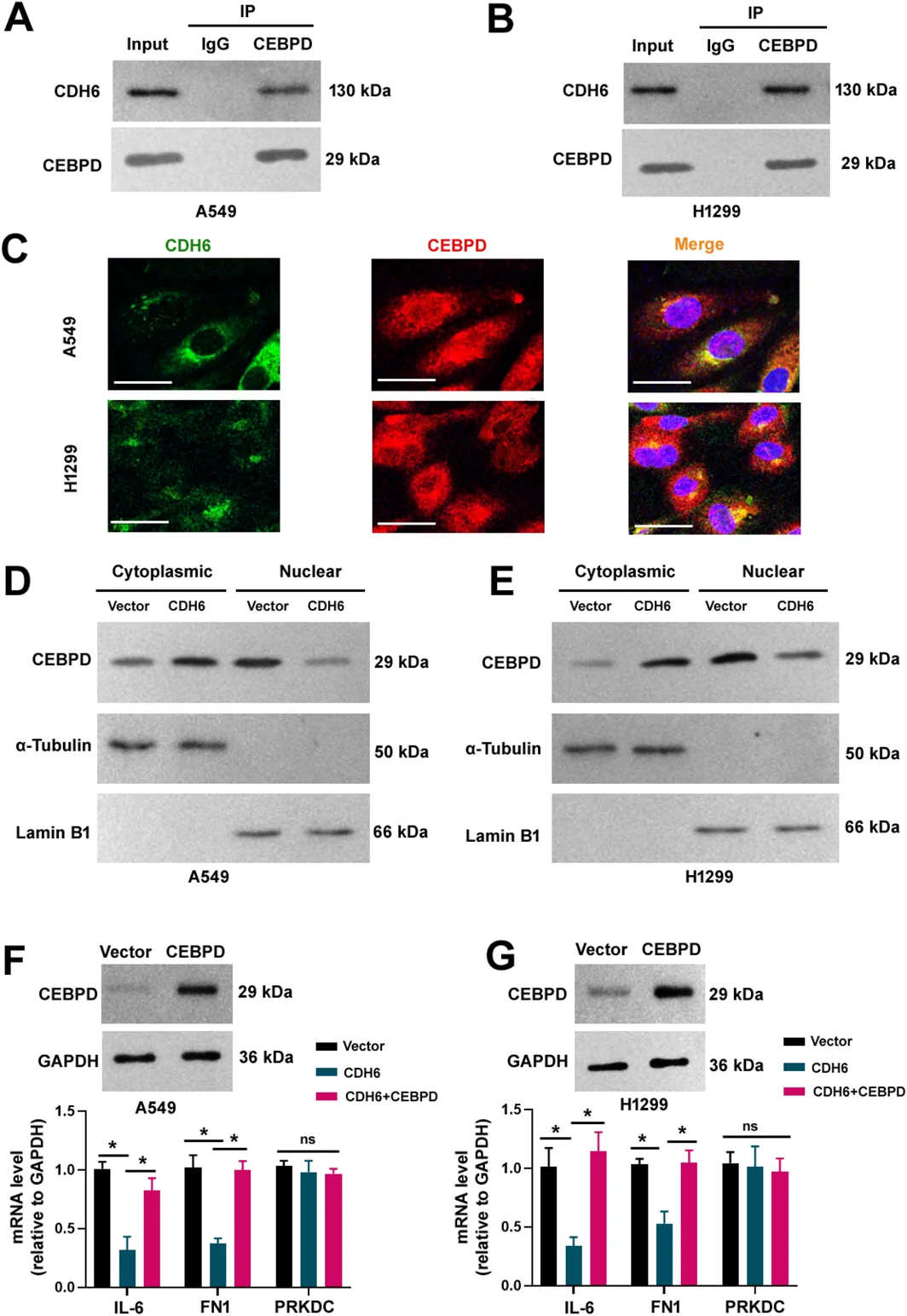
CDH6 interacts with CEBPD and reduces its nuclear localization. (A, B) Co‐immunoprecipitation was done using an anti‐CEBPD antibody to evaluate the interaction between CDH6 and CEBPD protein. (C) Colocalization analysis of CDH6 and CEBPD by immunofluorescence staining. Nuclei were counterstained with 4′,6‐diamidino‐2‐phenylindole (blue). Scale bar, 20 μm. (D, E) Western blot analysis was utilized to detect the nucleus and cytoplasm CEBPD expression after overexpression of CDH6. α‐Tubulin and lamin B1 were used as cytoplasmic and nuclear marker proteins, respectively. (F, G) Analysis of *IL‐6*, *FN1*, and *PRKDC* mRNA levels in A549 and H1299 cells transfected with the indicated constructs. Inserts show the overexpression of CEBPD in non‐small cell lung cancer cells after transfection with the CEBPD‐expressing plasmid. *p < 0.05 (*n* = 3). ns indicates no significance. CDH6, cadherin‐6; CEBPD, CCAAT enhancer binding protein delta.

To determine how CDH6 regulates CDDP sensitivity, we performed CDH6 immunoprecipitation in A549 cells and attempted to search for CDH6 partners. Mass spectrometry analysis of the proteins co‐immunoprecipitated with CDH6 revealed that CEBPD was among the top hits (Table S2). Co‐immunoprecipitation assay using anti‐CEBPD antibody validated the interaction between CDH6 and CEBPD (Figure 5A,B). Immunofluorescence staining further indicated that cytoplasmic CEBPD specifically colocalized with CDH6 in NSCLC cells (Figure 5C). CEBPD is known as a crucial transcription factor contributing to CDDP resistance in cancer cells.^26^ We speculated that the interaction between CDH6 and CEBPD might interfere with CEBPD nuclear localization and the expression of CEBPD target genes. To test this hypothesis, we overexpressed CDH6 in NSCLC cells and analyzed its impact on the subcellular distribution of CEBPD. Notably, overexpression of CDH6 raised the level of CEBPD in the cytoplasm but reduced the level of CEBPD in the nucleus (Figure 5D,E). A number of CEBPD target genes such as *PRKDC*,^26^ *IL‐6*,^27^ and *FN1* ^28^ have been found to be involved in CDDP resistance in human cancers.^29^, ^30^ Intriguingly, ectopic expression of CDH6 suppressed the expression of *IL‐6* and *FN1* rather than *PRKDC* in NSCLC cells (Figure 5F,G). Co‐expression of CEBPD restored the expression of *IL‐6* and *FN1* in CDH6‐overexpressing NSCLC cells (Figure 5F,G). Collectively, we demonstrate that CDH6 can sequester CEBPD and hamper its transcriptional activity in NSCLC cells.

### 3.6 CEBPD is required for CDH6 depletion‐induced CDDP resistance in NSCLC

**FIGURE 6:**
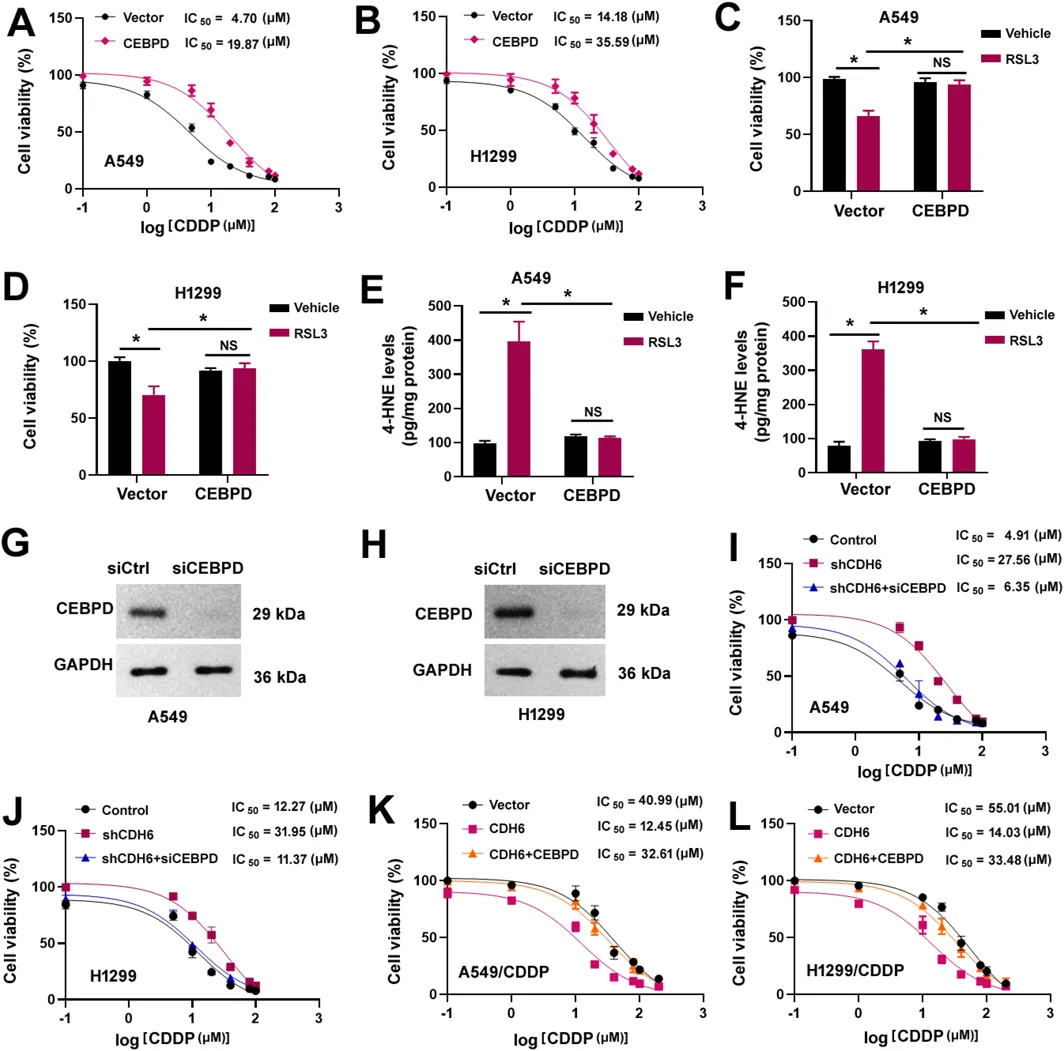
CEBPD is required for CDH6 depletion‐induced CDDP resistance in non‐small cell lung cancer. (A, B) Measurement of the IC_50_ of CDDP in A549 and H1299 cells transfected with the indicated constructs. (C, D) Assessment of cell viability after treatment with RSL3. *p < 0.05 (*n* = 3). ns indicates no significance. (E, F) Assessment of lipid peroxidation by quantifying 4‐hydroxynonenal levels. *p < 0.05 (*n* = 3). ns indicates no significance. (G, H) Western blot analysis was utilized to determine the efficiency of CEBPD knockdown in A549 and H1299 cells transfected with control siRNA (siCtrl) or a pool of 3 CEBPD‐targeting siRNAs (siCEBPD). (I, J) Measurement of the IC_50_ of CDDP in A549 and H1299 cells transfected with the indicated constructs. (K, L) Measurement of the IC_50_ of CDDP in A549/CDDP and H1299/CDDP cells transfected with the indicated constructs. CDDP, cisplatin; CDH6, cadherin‐6; siRNA, small interfering RNA; CEBPD, CCAAT enhancer binding protein delta.

Next, we validated the role of CEBPD in CDH6 deficiency‐induced CDDP resistance in NSCLC. Remarkably, overexpression of CEBPD conveyed resistance to CDDP in NSCLC cells (Figure 6A,B). Furthermore, CEBPD overexpression attenuated RSL3‐induced ferroptosis (Figure 6C,D) and lipid peroxidation (Figure 6E,F). Given the regulation of CEBPD nuclear localization by CDH6, we next checked if CDH6 deficiency‐induced CDDP resistance in NSCLC was mediated by CEBPD. To this end, we knocked down *CEBPD* in NSCLC cells using siRNA technology (Figure 6G,H). We found that depletion of *CEBPD* resensitized CDH6‐depleted NSCLC cells to CDDP (Figure 6I,J). Conversely, overexpression of CEBPD reversed the sensitization of CDDP‐resistant NSCLC cells by CDH6 (Figure 6K,L). Taken together, these results highlight the CEBPD dependence of CDH6‐depleted NSCLC cells in response to CDDP treatment.

### 3.7 The clinical significance of CEBPD upregulation in NSCLC

**FIGURE 7:**
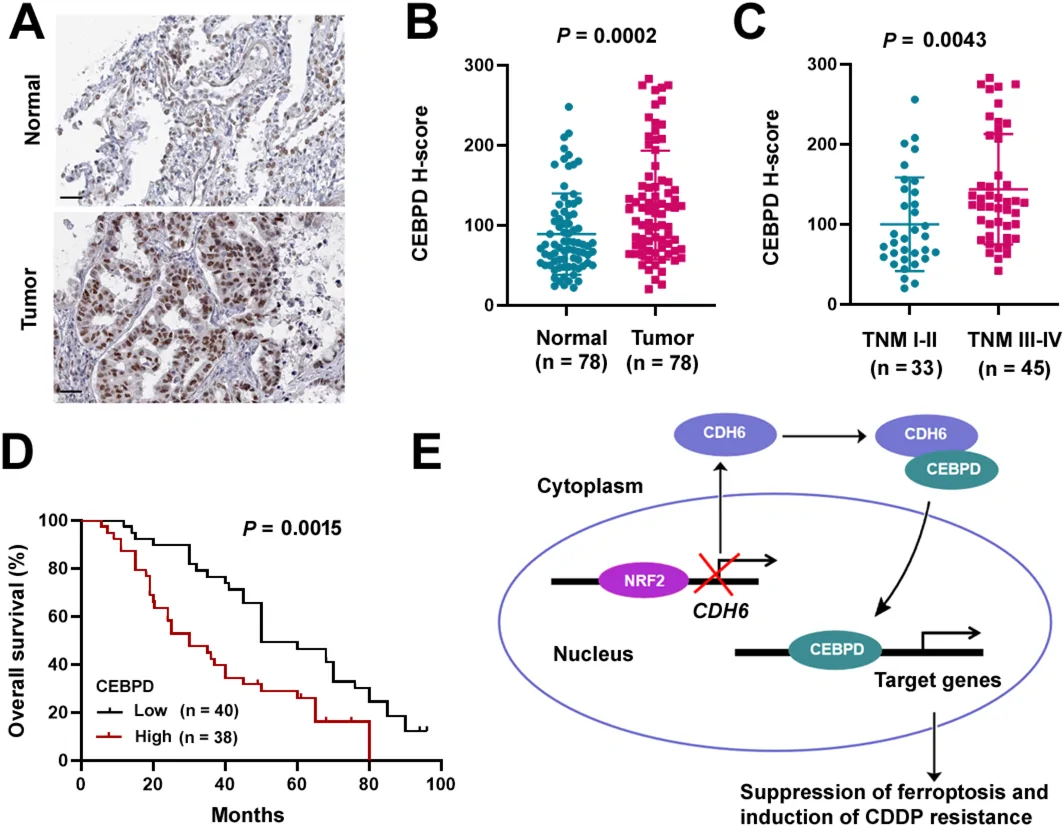
The clinical significance of CEBPD upregulation in NSCLC. (A) Representative images of CEBPD immunohistochemical staining in NSCLC and adjacent normal lung tissues. Scale bar, 100 μm. (B) Comparison of *H*‐scores for CEBPD staining in NSCLC and adjacent normal lung tissues. (C) Comparison of *H*‐scores for CEBPD staining in NSCLC samples at different stages. (D) Kaplan–Meier curves of overall survival according to CEBPD expression levels (high vs. low). (E) Proposed working model illustrating that nuclear factor erythroid 2‐related factor 2‐mediated downregulation of CDH6 modulates ferroptosis and CDDP resistance in NSCLC cells through the CEBPD‐dependent mechanism. CDDP, cisplatin; CDH6, cadherin‐6; CEBPD, CCAAT enhancer binding protein delta; NSCLC, non‐small cell lung cancer.

We further investigated the clinical relevance of CEBPD expression in NSCLC. High expression of CEBPD was observed in NSCLC tissues compared with paired normal tissues (*p* = 0.0002; Figure 7A,B). Moreover, NSCLC patients with advanced TNM stage showed significantly higher CEBPD expression (*p* = 0.0043; Figure 7C). Kaplan–Meier survival analysis indicated that the patients with high CEBPD expression had markedly shorter survival times than those with low CEBPD expression (*p* < 0.0015; Figure 7D). These data suggest that CEBPD upregulation correlates with poor prognosis in NSCLC.

## 4 DISCUSSION

In this study, we demonstrate *CDH6* as a novel tumor suppressor gene in NSCLC. CDH6 is downregulated in NSCLC tissues and associated with poor prognosis of NSCLC. Experimental results reveal that depletion of CDH6 enhances NSCLC cell tumorigenesis and CDDP resistance. Moreover, overexpression of CDH6 resensitizes CDDP‐resistant NSCLC cells to CDDP. Our data highlight the critical role of CDH6 in the regulation of NSCLC progression and chemoresistance (Figure 7E).

We show that the transcription factor NRF2 is involved in the dysregulation of CDH6 in NSCLC cells. There is a negative correlation between NRF2 and CDH6 in NSCLC tissues. Consistently, knockdown of NRF2 facilitates the expression of CDH6 in NSCLC cells. NRF2 is known to regulate a lot of genes involved in cellular oxidative stress response, contributing to NSCLC cell survival.^19^, ^31^ Unlike the positively regulated genes such as *ALDH3A1* ^32^ and *ABCF2*,^33^ CDH6 expression is suppressed by NRF2 activation. The promoter of the *CDH6* gene harbors a putative binding site for NRF2. ChIP assays confirm that NRF2 activation due to the sulforaphane treatment leads to the enrichment of NRF2 protein at the promoter of *CDH6*. The transcription factor RUNX2 has been shown to drive the transcription of *CDH6* in thyroid tumors.^24^ Consistently, our data indicated the induction of CDH6 expression by RUNX2 overexpression in NSCLC cells. Most importantly, the sulforaphane treatment blocked RUNX2‐induced transcription of *CDH6*. These results suggest that the NRF2 occupancy at the promoter of *CDH6* may prevent the RUNX2‐dependent transcription, consequently reducing CDH6 expression.

CDH6 may play a paradoxical role in tumor progression, exerting both tumor‐promoting and tumor‐suppressive effects.^17^, ^34^, ^35^ In a number of cancers, including ovarian and renal cancers, CDH6 interacts with integrins to promote cancer cell adhesion and invasion.^17^ However, in cholangiocarcinoma, *CDH6* is epigenetically silenced because of promoter hypermethylation and exhibits the ability to impede cancer cell growth.^35^ Our work demonstrates that CDH6 also plays a tumor‐suppressive role in NSCLC. Specifically, silencing of CDH6 increases the growth, colony formation, and tumorigenesis of NSCLC cells. Moreover, CDH6 depletion impairs the efficacy of CDDP against NSCLC cells. Gain‐of‐function experiments further indicate that overexpression of CDH6 overcomes CDDP resistance in CDDP‐resistant NSCLC cells. However, a previous study has reported that activated CDH6 expression is associated with the malignant behaviors of NSCLC cells induced by circular RNA circ_0000517.^36^ The conflicting results may be explained by different genetic contexts that can affect the functional outcomes of CDH6 upregulation in NSCLC.

It has been reported that induction of ferroptosis alleviates chemoresistance in cancer cells.^37^, ^38^ Targeting ferroptotic pathways is a potential strategy to improve anticancer treatments.^38^ Interestingly, CDH6 overexpression enhances ferroptotic response in CDDP‐resistant NSCLC cells, which is evidenced by enhancement of RSL3‐induced lipid ROS production and suppression of GPX4 expression. GPX4 is a master suppressor of ferroptosis and shows the ability to prevent lipid peroxidation.^25^ Thus, CDH6 may promote GPX4‐dependent ferroptosis in CDDP‐resistant NSCLC cells. Rescue experiments indicate that inhibition of ferroptosis using Lip‐1 or Fer‐1 attenuates CDH6‐induced sensitization of CDDP‐resistant NSCLC cells. These results indicate that CDH6‐mediated chemosensitization in NSCLC is ascribed to increased sensitivity to ferroptosis. Several members of the CDH family, such as CDH4^39^ and E‐cadherin^40^, have shown the ability to modulate ferroptosis. Our present data provide the first evidence for the regulation of ferroptosis by CDH6.

The biological activity of CDH6 mainly relies on the interaction with other protein partners such as integrins.^17^ In the present study, we performed CDH6 co‐immunoprecipitation assays to search for key protein partners in NSCLC cells. We found that CDH6 interacts with and sequesters CEBPD protein in the cytoplasm of NSCLC cells. These data suggest that CDH6 serves as a suppressor of CEBPD, reducing its nuclear entry and transcriptional activity in NSCLC cells. CEBPD plays a crucial role in cancer progression by regulating many target genes.^26^, ^27^, ^41^ For instance, CEBPD drives the transcription of *VAMP3* to promote paclitaxel resistance and immune evasion in triple‐negative breast cancer.^41^ CEBPD can transactivate *PRKDC* and enhance *PRKDC*‐mediated DNA damage repair in cervical cancer cells, thus causing CDDP resistance.^26^ However, our data showed that neither CDH6 nor CEBPD overexpression markedly modulated the expression of *PRKDC* in NSCLC cells. We then examined 2 other target genes of CEBPD, that is, *IL‐6* and *FN1*.^27^, ^28^ Of note, CDH6 overexpression increased the mRNA abundance of *IL‐6* and *FN1*, which was rescued by co‐expression of CEBPD. Both *IL‐6* and *FN1* have been documented to induce CDDP resistance in human cancers,^29^, ^30^ which partially explains the regulation of CDDP sensitivity by CEBPD.

In lung cancer, CEBPD has been reported to facilitate lymphangiogenesis and metastasis by regulating VEGF‐C autocrine signaling of endothelial cells.^42^ In this study, we explored the role of CEBPD in lung cancer cells and revealed that CEBPD overexpression triggers CDDP resistance and reduces ferroptosis. Most importantly, knockdown of *CEBPD* blocks *CDH6* silencing‐induced CDDP resistance in NSCLC cells. We also demonstrate that high expression of CEBPD is associated with aggressive parameters and poor prognosis in NSCLC patients. Therefore, our results support that CEBPD acts as an oncogene and mediates CDH6 deficiency‐induced CDDP resistance in NSCLC.

Although our current data indicate the interaction between CDH6 and CEBPD in NSCLC cells, further work is needed to map the interaction domains of CDH6 and CEBPD. Moreover, the downstream target genes mediating the CEBPD activity in NSCLC cells require further investigation. Additionally, there is a lack of validation of the clinical findings in a larger cohort of NSCLC patients. It is also important to evaluate the potential of CDH6 and CEBPD as independent prognostic factors in NSCLC patients.

In summary, NRF2‐mediated CDH6 downregulation potentiates CDDP resistance and prevents ferroptosis in NSCLC cells. The tumor‐suppressive activity of CDH6 is ascribed to the interaction with CEBPD, leading to the inhibition of CEBPD nuclear entry and then reduction of CEBPD‐dependent transcription. As a therapeutic target, CDH6 holds potential for improving CDDP‐based chemotherapy against NSCLC.

## AUTHOR CONTRIBUTIONS

Ri‐sheng Huang designed the study. Guang‐ze Zhou, Zhuo‐hang Zhu, Hong‐li Liao, Rui‐heng Chen, and Dan‐ni Wu performed the experiments and contributed to the analysis of data. Ri‐sheng Huang wrote the manuscript. All authors discussed the results and approved the manuscript.

## CONFLICT OF INTEREST STATEMENT

The authors declare no conflicts of interest.

## ETHICS STATEMENT

The study was approved by the Institutional Review Board of Wenzhou Medical University (Wenzhou, China). All patients provided written consent for the use of tissue samples for research. The animal experiments were approved by the Institutional Animal Care and User Committee of Wenzhou Medical University.

## Supporting information

Figure S1

Table S1

Table S2

## Data Availability

The data that support the findings of this study are available from the corresponding author upon reasonable request.
