# Supplementary material for: NRF2‐CDH6‐CEBPD axis suppresses ferroptosis and drives cisplatin resistance in non‐small cell lung cancer
Source: J Cell Commun Signal. 2026 Sep 23;20(3):e70115. doi: 10.1002/ccs3.70115 (PMC13601855; doi:10.1002/ccs3.70115)
Supplement: Supplementary file 1 [file CCS3-20-e70115-s001.docx]

**Supplementary data**

Supplementary Figure S1. The IC_50_ of cisplatin (CDDP) in CDDP-resistant and parental A549 and H1299 cells was assessed by the MTT assay.
