# Supplementary material for: NRF2‐CDH6‐CEBPD axis suppresses ferroptosis and drives cisplatin resistance in non‐small cell lung cancer
Source: J Cell Commun Signal. 2026 Sep 23;20(3):e70115. doi: 10.1002/ccs3.70115 (PMC13601855; doi:10.1002/ccs3.70115)
Supplement: Supplementary file 2 [file CCS3-20-e70115-s003.docx]

**Supplementary Table S1. Sequences of PCR primers and siRNAs**

| **Name** | **Sequence** 5′**-**3′ |
| --- | --- |
| **For quantitative PCR** |  |
| SOX4 | Forward: GAAGCTGCTCAAAGACAGCG |
|  | Reverse: CTGGGCCGGTACTTGTAGTC |
| GALNT13 | Forward: GAACGCTCTGGGTTAATACG |
|  | Reverse: CCCATAAGTCATGTCTGAC |
| CDH6 | Forward: AAGGAGGTTTACACAGCCACTGT |
|  | Reverse: CTTGGTACTGCTCCCTGTTTTCT |
| INPP4B | Forward: ACGCAGGAAAGTCAGGCTAA |
|  | Reverse: TGCCAGGTAACACCATTTCTT |
| PDE3A | Forward: GACAGCGATGAGTCAGGAGA |
|  | Reverse: TCTGAAGAGTGCGACTGAGG |
| PDK4 | Forward: CAAGATGCCTTTGAGTGTTCAA |
|  | Reverse: GGTCTTCTTTTCCCAAGACAAC |
| PLCB4 | Forward: GGCTACTTGAAGACACATGCA |
|  | Reverse: CTCATGAAATCTGGTTTGAGAA |
| CLDN4 | Forward: ATGCAGTGCAAGGTGTACGA |
|  | Reverse: CTTTCATCCTCCAGGCAGTT |
| RAP1GAP | Forward:CGACGAGGACAAGATGGAGA |
|  | Reverse: TGCTGGTGGACACGGTGTT |
| CFH | Forward:GGACACAGTGAAGTGGTGGAAT |
|  | Reverse: CCACAGGTACTCTCCTCCACA |
| NRF2 | Forward:TATCCATTCCTGAGTTACAGTGTC |
|  | Reverse: CTGTCAGTTTGGCTTCTGGAC |
| IL-6 | Forward:ACTCACCTCTTCAGAACGAATTG |
|  | Reverse: CCATCTTTGGAAGGTTCAGGTTG |
| FN1 | Forward: TGGAGACACCTGGAGCAAGAA |
|  | Reverse: GCTGCACGAACATCGGTGA |
| PRKDC | Forward: CAGAAGATCGCACCTTACTCTGT |
|  | Reverse: ACTTAATAAGAAGGTCCAGGGCT |
| GAPDH | Forward: CAGAACATCATCCCTGCCTCTAC |
|  | Reverse: TTGAAGTCAGAGGAGACCACCTG |
| **siRNAs** |  |
| siNRF2 | #1: CCAAAGAGCAGUUCAAUGA |
|  | #2: UAAAGUGGCUGCUCAGAAU |
|  | #3: UGACAGAAGUUGACAAUUA |
| siCEBPD | #1: GGCAGUUCUUCAAGCAGCU |
|  | #2: GCUGCAGUUUCUUGGGACA |
|  | #3: UGCAGCAGAAGUUGGUGGA |
